# Somatization Across Unconscious Conflict, Brain Circuits, and Culture

**DOI:** 10.1192/j.eurpsy.2026.11059

**Published:** 2026-07-31

**Authors:** E. M. Jetter, D. Valle, H. Nyugen, B. R. Carr

**Affiliations:** 1 College of Medicine; 2 Department of Psychiatry, University of Florida, Gainesville, United States

## Abstract

**Introduction:**

Rather than a vehicle for symbolism, psychoanalysis sees the body as an active site of conflict and expression. Freud first showed how repression can transform conflict into bodily symptoms (Freud S. Studies on Hysteria 1895). Building on this foundation, Alexander proposed the “specificity hypothesis,” linking emotional patterns to particular organ systems (Alexander F. Psychosomatic Medicine 1950). Expanding further, McDougall described the “theatre of the body,” where symptoms emerge when representation fails (McDougall J. Theatres of the Body 1989). Krystal deepened this perspective by emphasizing how deficits in affect regulation and alexithymia disrupt integration and self-healing (Krystal H. Integration and Self-Healing 1988). Extending these insights beyond the clinic, Kirmayer showed how idioms of distress vary across societies yet often find bodily expression (Kirmayer L. Culture and Somatization 1998). These perspectives show somatization as distress lived in the body when representation falters, a view that resonates today with research on limbic circuitry and with cross-cultural idioms of distress.

**Objectives:**

To explore how psychoanalytic theory can inform current views of somatization by linking unconscious processes with new findings in neuroscience and patterns of symptom expression across cultures.

**Methods:**

A narrative review included psychoanalytic writings, neuroimaging of medically unexplained symptoms, stress physiology, and cross-cultural studies of somatic presentations. It examined regressive behaviors and their impact on communication, adherence, and the therapeutic relationship.

**Results:**

Three complementary lenses illuminate the phenomenon of somatization. From a psychoanalytic perspective, symptoms embody unconscious conflict, reflect alexithymic difficulty in symbolizing affect, and enact what McDougall described as the “theatre of the body. Research on limbic–somatic circuitry and pain processing shows how arousal translates into bodily states, grounding persistence in physiology and meaning. From a cultural perspective, somatic idioms of distress function as socially legible forms of suffering, shaping how individuals communicate distress in ways that are recognized within families, communities, and clinical encounters. Viewed across these lenses, somatization is understood as the integration of psyche, brain, and culture.

**Conclusions:**

Somatization is best understood as a convergence zone: unconscious conflict, neurobiological stress pathways, and cultural expression. For medical education, this case shows why training should address not only ‘symptom management’ but also the meanings that body complaints carry across contexts.

**Disclosure of Interest:**

None Declared

